# Comparison between Endoscopic Ultrasound-Guided Antegrade and Transluminal Stent Implantation in Distal Malignant Biliary Obstruction after Failed ERCP

**DOI:** 10.1155/2024/1458297

**Published:** 2024-05-14

**Authors:** Yonghua Shen, Ying Lv, Xiaojiao Zheng, Wei Zhan, Senlin Hou, Lin Zhou, Jun Cao, Bin Zhang, Lei Wang, Hao Zhu, Lichao Zhang

**Affiliations:** ^1^Department of Gastroenterology, Nanjing Drum Tower Hospital, The Affiliated Hospital of Nanjing University Medical School, Nanjing, China; ^2^Biliopancreatic Endoscopic Surgery Department, Second Hospital of Hebei Medical University, Shijiazhuang, China

## Abstract

**Background:**

Distal malignant biliary obstruction (DMBO) can result in obstructive jaundice. Endoscopic ultrasound- (EUS-) guided biliary drainage (EUS-BD) has been an alternative for DMBO after failed ERCP.

**Aim:**

To compare the efficacy and safety between antegrade and transluminal approaches in patients with unresectable DMBO when ERCP failed.

**Methods:**

Patients with DMBO leading to obstructive jaundice after failed ERCP were enrolled in this study. We retrospectively evaluated the safety and efficacy between EUS-guided transluminal stenting (TLS group) and antegrade stenting (AGS group).

**Results:**

82 patients were enrolled, of which 45 patients were in TLS group and 37 in AGS group. There were no statistical differences in the malignancy type, baseline common bile duct diameter, total bilirubin level, reason for EUS-BD, and history of biliary drainage between TLS and AGS groups. The technical success rate was statistically higher in TLS group than in AGS group (97.8 vs. 81.1%, *P* = 0.031). There were no statistical differences in clinical success rate, procedure-related adverse events, stent migration rate, stent dysfunction rate, reintervention rate, and overall patient survival time between TLS and AGS groups. The median time to stent dysfunction or patient death in TLS and AGS groups was 53 and 81 days, respectively (*P* = 0.017).

**Conclusions:**

Although AGS had a lower technical success rate than TLS, it was superior to TLS in stent patency in patients with DMBO.

## 1. Introduction

Distal malignant biliary obstruction (DMBO) arises from various malignancies, which may result in obstructive jaundice because of the disturbed bile excretion. For several decades, unresectable DMBO usually undergoes endoscopic retrograde cholangiopancreatography (ERCP) accompanied by stent placement as the preferred palliative method with reasonable success rate and safety [1]. When ERCP cannot be successful, endoscopic ultrasound- (EUS-) guided biliary drainage (EUS-BD) has become the preferred option for bile duct bypass surgery or percutaneous transhepatic biliary drainage (PTBD) [2].

The EUS-BD technique includes transluminal (TL) biliary stent placement, EUS-guided antegrade transpapillary (or transanastomotic) stenting, and EUS-guided rendezvous (RV) approach [3–5]. Though the RV method can provide a physiologic anatomic pathway for drainage, it may fail due to inaccessible papilla or failure to advance guidewire through the stricture [6]. The TL technique should create a nonanatomic fistula linking the bile duct system with upper gastrointestinal tract, including hepatogastrostomy (HGS) and choledochoduodenostomy (CDS) approaches. Though the antegrade technique is appropriate for the patients with gastric outlet obstruction, it also needs to advance guidewire across the stricture and the papilla. Meanwhile, the antegrade approach usually needs additional drainage to reduce the risk of bile leakage. Compared with the TL approach, there is no sufficient evidence whether technical difficulties and increased costs can make the antegrade approach not to be the preference.

The purpose of our study was to compare technical and clinical success, adverse events, and survival between the two different EUS-guided drainage routes, antegrade or TL approach, in patients with unresectable DMBO when standard ERCP failed.

## 2. Patients and Methods

### 2.1. Patients

82 DMBO patients were treated by EUS-BD between December 2019 and February 2022 at the Drum Tower Hospital and the Second Hospital of Hebei Medical University. Enrolled patients with unresectable DMBO provided written consent to undergo possible EUS-BD. Before providing consents, all patients received necessary information about possible drainage approaches if ERCP failed. Histologic malignancy was proven, and unresectability was confirmed by preprocedure magnetic resonance imaging, computed tomography, or EUS in all patients. The inclusion criterion was the performance of EUS-guided transluminal stenting (TLS group) or EUS-guided antegrade stenting (AGS group) in DMBO patients after failed ERCP. Exclusion criteria included patients aged less than 18 years, resectable or borderline resectable lesions, large-volume ascites, bleeding diatheses, cholangitis, and poor cardiovascular condition. This retrospective study was conducted with the approval of the local research ethics committees.

### 2.2. Procedures

TL or antegrade stenting technique was selected at the discretion of the operators. TL technique was performed with only an echoendoscope. After biliary access, the puncture tract was dilated by various devices to facilitate antegrade stent placement. TL technique commonly entailed the creation of HGS or CDS. The successful performance of HGS is largely dependent on the ability to identify the enlarged left hepatic duct in segment 2 or 3 of the liver from the proximal body of the stomach or gastric remnant. With a linear array echoendoscope (GF-UCT260, Olympus, Tokyo, Japan), left hepatic duct was identified and punctured with a 19-gauge needle (Wilson Cook Medical, Bloomington, USA; and Boston Scientific, Natick, USA) with Color Doppler to avoid intervening vessels. After confirming bile aspiration, a cholangiogram was obtained with contrast injection. A hydrophilic 0.035 in./450 cm guidewire (Boston Scientific, Natick, USA) was advanced towards the biliary confluence. The needle was withdrawn, and the tract was then enlarged with a 6F cytostome (ENDO-FLEX GmbH, Germany) or Soehendra biliary dilation catheter (Wilson Cook Medical, Bloomington, USA) to permit stent placement.

CDS approach requires the creation of a neofistula between the common bile duct and duodenum. The target duct was identified ultrasonographically from the duodenal bulb. The bile duct access, tract enlargement, and stent placement were analogous to HGS.

**Figure 1:**
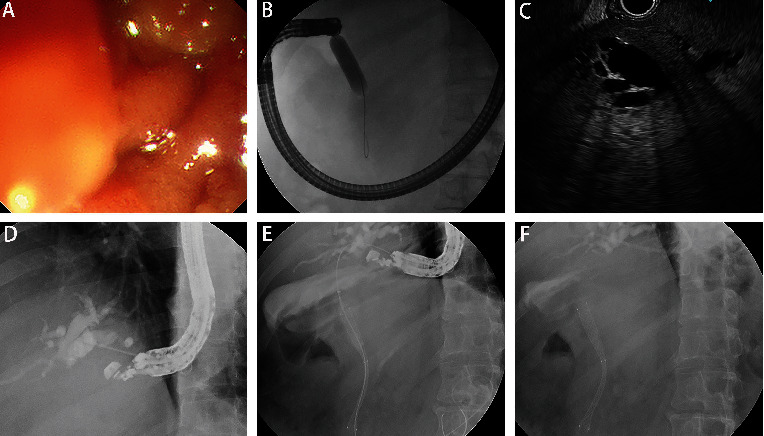
The procedure of antegrade stenting: (A) endoscopic view of the duodenal stricture; (B) X-ray image of stricture dilation with endoscope difficult to pass through subsequently; (C) endoscopic ultrasound-guided bile duct puncture pathway; (D) cholangiogram through endoscopic ultrasound-guided bile duct puncture; (E) X-ray image of released metal stent; (F) X-ray image of released metal stent with endoscopic nasobiliary drainage.

The initial steps of antegrade stenting technique for bile duct access, cholangiography, and tract enlargement were performed in a similar fashion to the TL approach. Unlike TL, the antegrade stent was advanced over the guidewire to traverse the stricture and then papilla or anastomosis (Figure 1).

### 2.3. Definitions

Technical success was considered as successful biliary drainage with plastic or metal stenting. Clinical success was considered a decrease in total bilirubin by at least 50% within 14 days of EUS-BD compared with the preprocedure level [7, 8]. Adverse events were defined as any clinically important events that required additional treatment including surgery, radiological intervention, endotherapy, medication, blood product transfusion, or prolongation of fasting period. After the biliary drainage, all patients were followed with adverse event assessment for 60 days [9]. Bleeding was diagnosed when any hemorrhagic event occurred and required blood transfusion, endoscopy, or prolongation of inpatient observation. Bile peritonitis was defined as newly emerged abdominal pain after the biliary drainage accompanied with elevation of the serum leukocyte or C-reactive protein level. Cholangitis was considered a new-onset fever with body temperature > 38°C at 24-48 h after the procedure accompanied with persistent abnormal liver function. Stent migration was considered as the necessity to retrieve the stent in the enteral lumen or biliary duct. Stent dysfunction was diagnosed if the patient suffered recurrent cholangitis originated from stent occlusion because of sludge in plastic stent, or tissue overgrowth, ingrowth, or sludge in metal stent. Reintervention was considered as any type of additional percutaneous, endoscopic, or surgical intervention to improve biliary drainage with dilated biliary tract on radiological imaging. Median survival time was assessed for the follow-up period and delineated with Kaplan-Meier analysis.

### 2.4. Statistical Analysis

Statistical analyses were performed using the software package SPSS 22.0. Continuous variables in different groups were compared with Student's *t* test or Wilcoxon rank-sum test. Categorical variables were evaluated by chi-squared or Fisher's exact test as appropriate. *P* value of <0.05 was considered significantly different.

## 3. Results

Patient demographic and clinical information are summarized in Table 1. Overall, 38 females and 44 males were included in the study, of which 45 patients were in TLS group and 37 in AGS group. The reasons for EUS-BD included pancreatic cancer (12 patients in TLS and 11 in AGS group), ampullary cancer (7 patients in TLS and 8 in AGS group), distal cholangiocarcinoma (7 patients in TLS and 5 in AGS group), metastatic adenopathy (9 patients in TLS and 7 in AGS group), duodenal cancer (6 patients in TLS and 4 in AGS group), and gallbladder cancer (4 patients in TLS and 2 in AGS group). There was no statistical difference in malignancy type between TLS and AGS groups (*P* = 0.963). All the patients had intra- and extrahepatic biliary dilation. There was no statistical difference in common bile duct diameter between TLS and AGS groups (12.5 ± 4.8 mm vs. 10.6 ± 5.4 mm, *P* = 0.143). In addition, the baseline total bilirubin level was not different between the two groups (TLS vs. AGS group, 238.4 ± 118.5 mg/dL vs. 191.4 ± 99.0 mg/dL; *P* = 0.102).

The reasons for EUS-BD included surgically altered anatomy (11 patients in TLS and 10 in AGS group), gastric outlet obstruction (25 patients in TLS and 19 in AGS group), and biliary cannulation failure (9 patients in TLS and 8 in AGS group). There was no statistical difference in the reason for EUS-BD between TLS and AGS groups (*P* = 0.930). Because of gastric outlet obstruction, previous enteral stent placement (8 patients in TLS and 4 in AGS group, *P* = 0.374) and EUS-guided gastroenterostomy (9 patients in TLS and 4 in AGS group, *P* = 0.257) have been performed without statistical differences. There was no significant difference in the history of biliary drainage between the two groups (*P* = 0.495).

As shown in Table 2, technical success rate was statistically higher in TLS group than in AGS group (97.8% vs. 81.1%, *P* = 0.031). In both groups, 7F plastic stent was placed with appropriate length. In TLS group, stent deployment by the CDS approach was failed in a patient. In AGS group, guidewire failed to traverse the stricture in 7 patients, although the subsequent TLS approach succeeded in all patients. Clinical success was achieved in 37 patients (84.1%) in TLS and 27 (90.0%) in AGS group. There was no statistical difference in clinical success rate between TLS and AGS groups (*P* = 0.701). In TLS group, 9 patients had procedure-related adverse events including bile peritonitis (5 patients, 11.1%), bleeding (1 patient, 2.2%), and cholangitis (3 patients, 6.7%). In AGS group, 5 patients had procedure-related adverse events including bile peritonitis (3 patients, 8.1%) and cholangitis (2 patients, 5.4%). No statistical difference was found in the overall procedure-related adverse events rate between TLS and AGS groups (20.0% vs. 13.5%, *P* = 0.627). PTBD was needed in 2 patients with stent migration in TLS group. In AGS group, the patients with stent migration were managed conservatively. There was no statistical difference in stent migration rate between TLS and AGS groups (*P* = 0.821). The stent dysfunction rate was also not statistically different between TLS and AGS groups (52.3% vs. 63.3%, *P* = 0.346). Reintervention was needed in 8 (18.2%) and 6 (20.0%) patients in TLS and AGS groups, respectively (*P* = 0.845). The approaches for reintervention included PTBD, ERCP, and stent exchange through previous puncture passage. In TLS group, 1 patient underwent PTBD, 1 patient underwent ERCP, and the other 6 patients underwent stent exchange through previous puncture passage. In AGS group, 4 patients underwent PTBD and 2 patients underwent ERCP.

**Figure 2:**
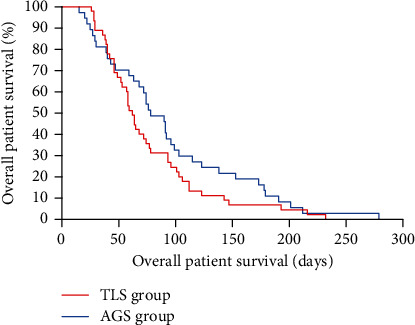
The Kaplan-Meier curves for the overall patient survival time in TLS and AGS groups in patients with distal malignant biliary obstruction. The median survival time of patients in TLS and AGS groups was 62 and 78 days, respectively (*P* = 0.190, log-rank test).

**Figure 3:**
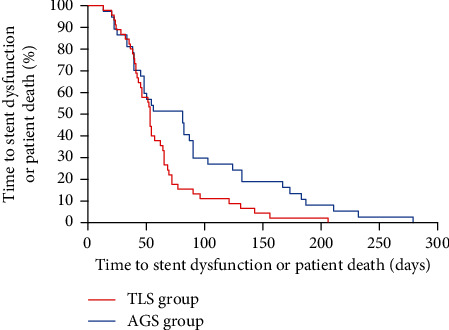
The Kaplan-Meier curves for the median time to stent dysfunction or patient death in TLS and AGS groups in patients with distal malignant biliary obstruction. The median time to stent dysfunction or patient death in TLS and AGS groups was 53 and 81 days, respectively (*P* = 0.017, log-rank test).

At the time of retrospective assessment (March 22, 2023), all patients had died. Figures 2 and 3 exhibit the Kaplan-Meier curves for overall survival time and the periods to stent dysfunction or individual death in TLS and AGS groups among DMBO individuals. The median survival time in TLS and AGS groups was 62 and 78 days, respectively (*P* = 0.190). There was no statistical difference in the aspect of overall patient survival between the two groups. The median time to stent dysfunction or patient death in TLS and AGS groups was 53 and 81 days, respectively (*P* = 0.017).

## 4. Discussion

After failed ERCP, HGS has the widest application against the effects of duodenal obstruction or difficult biliary strictures in EUS-BD procedures. The previous data showed that HGS and CDS approaches for EUS-BD were similarly safe and effective [10]. DMBO allows mainly CDS or a RV maneuver. Limited data demonstrated no difference in success and complication compared to transpapillary or transanastomotic access with TL access [11]. Obviously, EUS-AGS or EUS-RV is more suitable to the physiological requirements. However, gastric outlet obstruction and surgically altered anatomy limit the RV technique because of the antegrade guidewire manipulation difficulties. EUS-guided TL biliary drainage combined with EUS-AGS using metal stents has been shown to be effective and safe in 39 patients with malignant biliary obstruction [12]. Compared with HGS, AGS with HGS revealed a preference in terms of stent patency and safety in malignant biliary stricture patients receiving chemotherapy, although the technical success of AGS with HGS was inferior to that of HGS [13]. However, as partially stated in the article, this study also has some limitations. First, the conclusions may not apply universally because the study was performed at a single center. Second, this study did not include CDS in TL approach or simultaneously in EUS-AGS. Thirdly, the study did not address the reconstruction of the gastric outlet, which might have an effect on bile drainage. Thus, we performed a retrospective multicenter research to compare technical and clinical success, adverse events, and survival between EUS-AGS and EUS-TLS in patients with unresectable DMBO after ERCP failed.

The antegrade approach may have an advantage with respect to stent patency. On the one hand, metal stents may maintain patency longer than plastic stents, although metal stents at tumor sites may face the problems with tissue ingrowth. On the other hand, when placing a metal stent, a plastic stent will also be placed, which can play the role of “double insurance.” When one stent occludes, the other can offer an outlet to avoid reintervention. A previous study demonstrated that acute pancreatitis occurred in 8.3% of patients after AGS intervention [14]. The stent deployed at major papilla without sphincterotomy may increase the incidence of acute pancreatitis because of the pancreatic duct orifice compression. However, in our study, there was no acute pancreatitis in the AGS group with uncovered metal stent placement. The presence of metal mesh ensured that the pancreatic duct opening would not be blocked, confirming that the placement of uncovered metal stents was safe in AGS. Inconsistent with the previous study [13], the rates of clinical success and complication did not differ significantly between AGS and TLS. We speculate that it is related to the release of gastrointestinal pressure influenced by enteral stent placement or EUS-guided gastroenterostomy. The antegrade approach may keep away from gastric outlet obstruction without affection of the pressure. In this study, the comparatively high rates of enteral stent placement and EUS-guided gastroenterostomy may offer advantages in the improvement of efficacy and safety of TLS by alleviation of gastrointestinal pressure. There are differences between DMBO and hilar cholangiocarcinoma in terms of drainage methods, adverse events, and prognosis. This study only included distal cholangiocarcinoma to avoid heterogeneity of the patients, which was also different from previous studies.

In this study, there were some limitations. First, the retrospective nature of this study might result in potential biases in patient selection to limit the validity of conclusions. Second, our study also had a small sample size, despite performing in two centers and setting a control group. Therefore, more prospective, randomized controlled studies between both groups are needed to confirm the results.

In brief, this study shows that EUS-AGS has similar short-term efficacy and safety compared with TLS. Though technical success was inferior in AGS, it might be preferred in DMBO patients because of longer stent patency.

## Figures and Tables

**Table 1:** Patient demographic and clinical characteristics.

	TLS group (*n* = 45)	AGS group (*n* = 37)	*P* value
Age, mean ± SD (range), years	59.9 ± 11.8	63.4 ± 11.6	0.256
Sex, *n*			0.204
Male	27	17	
Female	18	20	
Type of malignancy			0.963
Pancreatic cancer	12	11	
Ampullary cancer	7	8	
Distal cholangiocarcinoma	7	5	
Metastatic adenopathy	9	7	
Duodenal cancer	6	4	
Gallbladder cancer	4	2	
Baseline total bilirubin (mg/dL)	238.4 ± 118.5	191.4 ± 99.0	0.102
Common bile duct diameter (mm)	12.5 ± 4.8	10.6 ± 5.4	0.143
Reason for EUS-guided biliary drainage			0.930
Surgically altered anatomy	11	10	
Gastric outlet obstruction	25	19	
Biliary cannulation failure	9	8	
History of biliary drainage	10 (22.2%)	6 (16.2%)	0.495
Previous enteral stent placement	8 (17.8%)	4 (10.8%)	0.374
Previous EUS-guided gastroenterostomy	9 (20.0%)	4 (10.8%)	0.257

**Table 2:** Comparison of outcome measures in the patients.

	TLS group (*n* = 45)	AGS group (*n* = 37)	*P* value
Technical success	44 (97.8%)	30 (81.1%)	0.031
Clinical success	37 (84.1%)	27 (90.0%)	0.701
Adverse events			0.627
Bile peritonitis	5	3	
Bleeding	1	0	
Cholangitis	3	2	
Stent migration	2 (4.5%)	3 (10.0%)	0.821
Stent dysfunction	23 (52.3%)	19 (63.3%)	0.346
Need for reintervention	8 (18.2%)	6 (20.0%)	0.845

## Data Availability

The data used to support the findings of the study are available from the corresponding authors on request.
